# *Ex vivo* Hsp70-Activated NK Cells in Combination With PD-1 Inhibition Significantly Increase Overall Survival in Preclinical Models of Glioblastoma and Lung Cancer

**DOI:** 10.3389/fimmu.2019.00454

**Published:** 2019-03-22

**Authors:** Maxim Shevtsov, Emil Pitkin, Alexander Ischenko, Stefan Stangl, William Khachatryan, Oleg Galibin, Stanley Edmond, Dominik Lobinger, Gabriele Multhoff

**Affiliations:** ^1^Radiation Immuno-Oncology, Center for Translational Cancer Research, TUM (TranslaTUM), Munich, Germany; ^2^Institute of Cytology of the Russian Academy of Sciences (RAS), St. Petersburg, Russia; ^3^Pavlov First Saint Petersburg State Medical University, St. Petersburg, Russia; ^4^Almazov National Medical Research Centre, Polenov Russian Scientific Research Institute of Neurosurgery, St. Petersburg, Russia; ^5^Wharton School, University of Pennsylvania, Philadelphia, PA, United States; ^6^Research Institute of Highly Pure Biopreparations, St. Petersburg, Russia

**Keywords:** membrane Hsp70, glioblastoma, lung carcinoma, immunophenotyping, NK cell therapy, anti-PD-1 antibody

## Abstract

Heat shock protein 70 (Hsp70) which is expressed on the plasma membrane of highly aggressive tumors including non-small cell lung carcinoma and glioblastoma multiforme serves as a target for Hsp70-targeting NK cells. Herein, we aimed to investigate the antitumor effects of a combined therapy consisting of *ex vivo* Hsp70-peptide TKD/IL-2-activated NK cells in combination with mouse/human anti-PD-1 antibody in a syngeneic glioblastoma and a xenograft lung cancer mouse model. Mice with membrane Hsp70 positive syngeneic GL261 glioblastoma or human xenograft A549 lung tumors were sham-treated with PBS or injected with *ex vivo* TKD/IL-2-activated mouse/human NK cells and mouse/human PD-1 antibody either as a single regimen or in combination. Tumor volume was assessed by MR scanning and tumor-infiltrating CD8^+^ T, NK, and PD-1^+^ cells were quantified by immunohistochemistry (IHC). We could show that the adoptive transfer of *ex vivo* TKD/IL-2-activated mouse NK cells or the inhibition of PD-1 resulted in tumor growth delay and an improved overall survival (OS) in a syngeneic glioblastoma mouse model. A combination of both therapies was well-tolerated and significantly more effective with respect to both outcome parameters than either of the single regimens. A combined treatment in a xenograft lung cancer model showed identical effects in immunodeficient mice bearing human lung cancer after adoptive transfer of TKD/IL-2-activated human effector cells and a human PD-1 antibody. Tumor control was associated with a massive infiltration with CD8^+^ T and NK cells in both tumor models and a decreased in PD-1 expression on immune effector cells. In summary, a combined approach consisting of activated NK cells and anti-PD-1 therapy is safe and results in a long-term tumor control which is accompanied by a massive tumor immune cell infiltration in 2 preclinical tumor models.

## Introduction

Stress-inducible Hsp70 is frequently overexpressed in the cytosol of many tumor entities where it fulfills a large variety of chaperoning functions such as folding/unfolding and transport of other proteins (1). Furthermore, highly aggressive tumors including glioblastoma (2–4) and lung cancers (5) present Hsp70 on their plasma membrane as a tumor-specific biomarker. Membrane Hsp70 positive, viable tumor cells have been found to actively release Hsp70 in exosomes, and therefore elevated exosomal Hsp70 levels in the serum are predictive for viable tumor mass (5). Increased Hsp70 membrane densities are detectable in highly aggressive tumors including primary glioblastoma multiforme (2) and advanced non-small cell lung cancer (NSCLC) (6). Both tumor types are debilitating, life-threatening diseases with poor prognosis. Despite combined treatment regimens consisting of surgery, radiotherapy (RT) and chemotherapy, OS and local progression-free survival (LPFS) in patients with glioblastoma multiforme and NSCLC in stage IIIA/B remains poor with < 15 months (7–9). In preclinical tumor models, radio-chemotherapy (RCT) has been found to induce abscopal effects (10–13), however, due to anti-apoptotic pathways and immunosuppressive mechanisms (14) these bonafide immunostimulatory effects are unable to mediate long-term protective anti-tumor immunity (15). A major breakthrough has been achieved by the application of immune checkpoint inhibitor antibodies which provide inhibitory feedback loops for an immune cell mediated tumor rejection (16). Many cancer types including brain and lung tumors use the PD-1 pathway for immune escape (17). Nivolumab, a fully humanized IgG_4_ antibody, targets PD-1 and thereby attenuates inhibitory signals in immune cells such as T and NK cells (16, 18), which results in objective tumor responses predominantly in highly immunogenic (“hot”) tumors (19, 20). Despite these promising results a relevant proportion of patients, however, does not profit from immune checkpoint inhibitor blockade therapies. Therefore, herein a combined regimen consisting of Hsp70-targeting activated NK cells and anti-PD-1 inhibition was tested in a preclinical syngeneic glioblastoma and a xenograft lung cancer model.

## Materials and Methods

### Cells

The mouse glioblastoma cells line GL261, human lung carcinoma A549 cells (American type culture collection (ATCC #CCL-185) and the NK target cell line K562 (ATCC #CCL-243) were cultured in Roswell park Memorial Institute 1640 medium supplemented with 10% (v/v) heat-inactivated fetal calf serum (FCS), 2 mM L-glutamine, 1 mM sodium pyruvate, and antibiotics (100 IU/mL penicillim, 100 μg/mL streptomycin) at 37°C in 95% humidity and 5% (v/v) CO_2_. Lewis lung carcinoma (LLC) cells were cultured in DMEM medium supplemented with 10% FCS, 2 mM L-glutamine and antibiotics (100 IU/mL penicillin, 100 μg/mL streptomycin). All cell lines are positive for membrane-bound Hsp70 as determined by flow cytometry (21, 22).

### Animals

C57Bl/6 male 10-week-old mice were purchased from the animal nursery “Rappolovo” of the Russian Academy of Medical Sciences (St. Petersburg, Russia). NMRI nu/nu 8–10-week male mice were obtained from an animal breeding colony (Charles River). All animal experiments were approved by the local ethical committee of Pavlov First St. Petersburg State Medical University (St. Petersburg, Russia) and were in accordance with institutional guidelines for the welfare of animals.

### Orthotopic Injection of GL261 Glioblastoma Cells Into C57Bl/6 Mice

Briefly, C57BL/6 mice were anesthetized by ip injection with fentanyl (0.05 mg/kg), midazolam (5 mg/kg) and medetomidine (0.5 mg/kg) mixture before mounting them in a stereotactic frame (David Kopf Instruments, Tujunda, CA, USA). GL261 cells (1 × 10^5^) resuspended in sterile PBS (2 μl) were stereotactically injected into the *nucleus caudatus dexter* of anesthetized mice.

### Orthotopic Injection of A549 Lung Cancer Cells Into Immunodeficient Mice

After anesthesia, NMRI nu/nu mice were injected percutaneously in the upper margin of the sixth rib on the right anterior axillary line into the right lung (5 mm depth) with a single cell suspension (100 μl) of A549 cells (5 × 10^6^ cells/ml).

### *Ex vivo* Stimulation of Mouse/Human NK Cells With TKD/IL-2

Peripheral blood lymphocytes (PBLs) were isolated of sacrificed C57BL/6 mice by Ficoll-Paque gradient centrifugation. After separation, PBL were resuspended in RPMI-1640 supplemented with 2 mM L-glutamine, 10% FCS, and antibiotics (100 IU/ml Penicillin G and 100 μg/ml Streptomycin). Previous data have indicated that NK cell activation is superior when, instead of purified NK cells, PBL are stimulated with the 14-mer TKD peptide (TKDNNLLGRFELS, 2 μg/ml, Bachem, Bubendorf, Switzerland) and IL-2 (100 IU/ml) at defined cell densities of 5–10 × 10^6^ PBL/ml for 3–4 days (23, 24). Since the human TKD sequence differs only in one amino acid in human and mouse (TKDNNLLGRFELSG and TRDNNLLGRFELSG, respectively), it is possible to stimulate mouse NK cells with the human TKD peptide (4).

Human PBL for NK cell stimulation for the treatment of the A549 xenograft tumor mouse model were obtained from Caucasian healthy volunteers (age range 22–24 year, age mean 23.1 years). All healthy individuals who participated in this study provided written informed consent. The study was approved by the local ethical committee.

Ten ml of peripheral blood was collected into EDTA tubes and PBL were isolated by density gradient centrifugation using Ficoll-Paque, as described earlier. After separation, PBL were resuspended in RPMI-1640 supplemented with 2 mM L-glutamine, 10% FCS, and antibiotics (100 IU/ml Penicillin G and 100 μg/ml Streptomycin). PBL were stimulated either with the 14-mer TKD peptide (TKDNNLLGRFELS, 2 μg/ml, Bachem, Bubendorf, Switzerland) or recombinant, low-endotoxin Hsp70 protein (10 μg/ml) that was obtained and purified from bacteria transformed with a pMSHSP plasmid, as described previously (23), and IL-2 (100 IU/ml) at cell densities of 5–10 × 10^6^ PBL/ml for 3−5 days (24, 25). Flow cytometry was performed on day 5 after stimulation with TKD/IL-2 using FITC/PE/PerCP or APC conjugated mouse IgG1 antibodies (BD Biosciences), FITC-conjugated mouse antibody against CD94 (BD Pharmingen), FITC/PE or APC conjugated mouse antibodies against CD56 (BD Biosciences), PerCP conjugated antibody against CD3 (BD Biosciences), FITC conjugated antibody against CD4 (BD Pharmingen), FITC or PE conjugated antibodies against CD8 (BD Pharmingen), PE conjugated antibody against CD19 (BD Pharmingen), PE conjugated antibody against CD16 (BD Pharmingen), PE conjugated monoclonal antibodies against NK cell activatory receptors (NKG2D (R&D Systems), NKp30 (Beckman Coulter), NKp46 (Beckman Coulter), APC-conjugated antibodies against CD45 (Life Technologies) and CD69 (BD biosciences). The percentage of positively stained cells was determined following subtraction of cell stained with an isotype-matched negative control antibody. Only PI (propidium iodide, Sigma) negative, viable cells were gated and analyzed.

### Cytotoxicity Assay

GL261, A549, and LLC cells and K562 cells were employed as target cells for analysis of the cytolytic activity of NK cells. The effector cells were isolated from C57/Bl6 mice (for GL261 and LLC cells) and peripheral blood of healthy individuals (for human A549 adenocarcinoma cells). Target cells were treated as follows: (1) control; (2) NK cells following co-incubation with IgG isotype antibody (20 μg/ml); (3) NK cells co-incubated with mouse/human anti-PD-1 immune checkpoint inhibitor antibody (20 μg/ml); (4) NK cells without stimulation; (5) NK cells *ex vivo* TKD/IL-2-stimulated (2 μg/ml for TKD peptide and 100 IU/ml for IL-2); (6) NK cells *ex vivo* TKD/IL-2-stimulated in combination with anti-PD-1 immune checkpoint inhibitor antibody (20 μg/ml). The incubation of the effector and target cells at various ratios (1:12.5, 1:25, and 1:50) lasted 4 h. CytoTox 96® non-radioactive cytotoxicity assay (Promega, USA) was employed to determine the amount of dying target cells according to the manufacture's protocol.

### Treatment Protocol

For comparing the efficacy of singular or combined therapies consisting of an adoptive transfer of *ex vivo* TKD/IL-2-stimulated mouse/human NK cells and mouse/human anti-PD-1 immune checkpoint inhibitor antibody (RMP1-30, eBioscience, Frankfurt/Main, Germany) animals with comparable tumor sizes (according to MRI volumometrics) were randomly divided into 5 groups (8 animals per group): Animals of the control groups were injected either with 100 μl PBS (iv) or with 250 μg isotype-matched IgG antibody (ip) on days 6, 9, 12 and 15. Animals of the treatment groups were iv injected either with NK cells (6 × 10^6^ in 100 μl PBL) on days 6, 9, and 12 and/or ip injected with anti-PD-1 antibody on days 6 (500 μg), 9 (250 μg), 12 (250 μg), and 15 (250 μg) in a volume of 500 μl PBS.

### Magnetic Resonance (MR) Tumor Imaging of Mouse Glioblastoma

Tumor progression was assessed before and after each therapy on days 5, 10, 15, 20, 25, and 30 using a high-field 11.0 T MR scanner (Bruker, Bremen, Germany) with a customized rodent coil. High-resolution anatomical T_2_-weighted scans (repetition time [TR]/echo time [TE] 4,200/36 ms, flip angle 180°, slice thickness 1.0 mm, interslice distance 1.2 mm, field of vision (FoV) 3.0 × 3.0 cm, matrix 256 × 256, in total 20 slices) were performed in coronal planes. Additionally T_1_-weighted scans (TR/TE 1500/7.5 ms, flip angle 180°, slice thickness 1.0 mm, FoV 3.0 × 3.0 cm, matrix 256 × 256), FLASH scans (TR/TE 350/5.4 ms, flip angle 40°, slice thickness 1.0 mm, 3.0 × 3.0 cm, matrix 256 × 256) in coronal planes were performed. The obtained images were analyzed using adequate software (AnalyzeDirect Inc, Overland Park, KS, USA).

### Mouse Tumor Immunohistochemistry (IHC)

Animals were anesthetized by ip injection of 150–200 mg/kg pentobarbital. After perfusion with 100 ml saline/4% paraformaldehyde, whole brains were removed and tumor volumes were assessed. Tissue was fixed in 4% paraformaldehyde/30% sucrose, embedded in Tissue-Tek® and blocks were cut into serial sections (5–7 μm). CD8^+^ T cells, NK1.1^+^ cells and PD-1^+^ lymphocytes were stained on IHC sections using anti-CD8 (53-6.7, Biolegend, San Diego, CA, USA), anti-NK1.1 (PK136, Biolegend, San Diego, CA, USA) and anti-PD-1 (RMP1-30, eBioscience, Frankfurt/Main, Germany) antibodies according to an established protocol. Tumor-infiltrating CD8^+^ T cells, NK1.1 cells and PD-1^+^ cells were counted in 3 fields of views by two independent researchers.

### Human Tumor Immunohistochemistry (IHC)

For IHC formalin-fixed, paraffin-embedded (FFPE) specimens of the A549 lung tumors were cut at 4 μm and transferred onto slides. All staining procedures were automatically performed on a Ventanas Benchmark XT for analysis of tumor-infiltrating CD8^+^, PD-1^+^, and CD56^+^ cells.

### Statistics

The comparative survival of animals was assessed with Kaplan-Meier curves that are based on the Kaplan-Meier estimator. All such estimates were computed and visually presented with corresponding confidence intervals. The Kaplan-Meier estimator is a non-parametric statistic that accommodates right-censoring in the data. When the means of the groups of two continuous variables were compared, the parametric Student's *t*-test was employed. Variances between groups were not considered to be equal, and degrees of freedom for such tests were computed accordingly. The significance level for all tests was alpha = 0.05, and all confidence intervals are reported at the 95% level. All p-values reported for all t-tests are two-sided. When comparing multiple groups, each of which had so few observations that standard parametric assumptions could not be validated, the Kruskal-Wallis test, which is a non-parametric analog to the one-way ANOVA test, was applied. The Krukal-Wallis test analyzes the differences in ranks between groups, rather than the difference in means. Depending on the test, either Statistica Version 9.2 for Windows or the R programming language was run for all tests. All experiments were conducted once on each animal.

## Results

### Analysis of the Phenotype of Human NK Cells After Stimulation With TKD/IL-2

**Figure 1:**
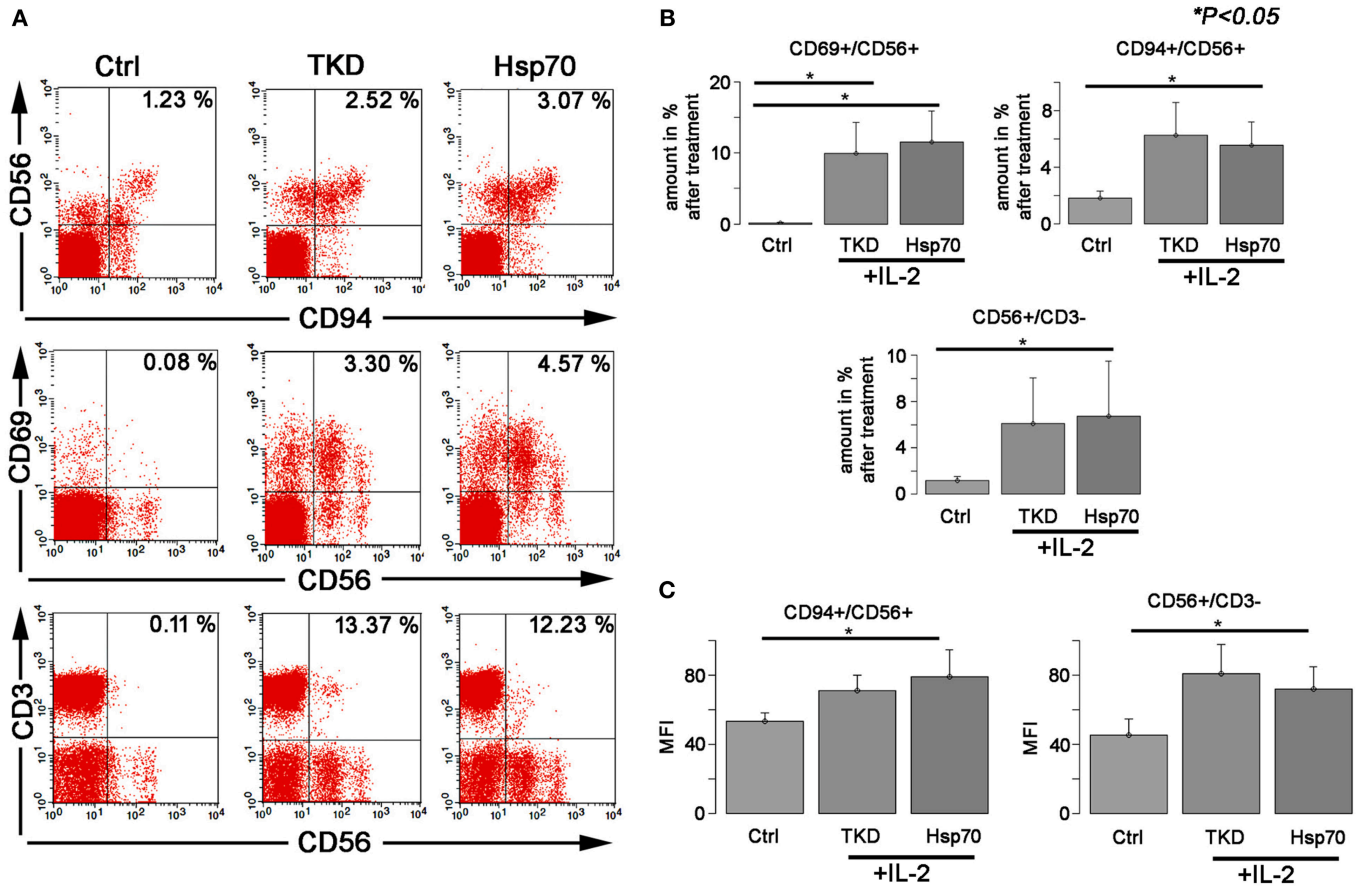
The effect of TKD/IL-2 or Hsp70/IL-2 on the expression of CD56, CD94, and CD69 receptors in peripheral blood lymphocytes (PBLs) of healthy individuals. The expression of the receptors was examined within the fraction of stimulated and unstimulated PBLs derived from 10 healthy Caucasian individuals. **(A)** Representative figure of the healthy individual following stimulation with TKD/IL-2 or Hsp70/IL-2. **(B)** The amount and **(C)** the median fluorescence intensity (MFI) of the receptors expressed on NK cells. Data presented as means ± standard error (M ± SE).

Compared to unstimulated cells, a treatment with TKD/IL-2 for 5 days results in a significant upregulation of CD94, CD69, and CD56 on CD3-negative, human NK cells (Figures 1A,B). The percentage of CD94^+^ cells increased from 1.83 ± 0.48 to 6.27 ± 2.31%, that of CD69^+^ cells from 0.14 ± 0.09 to 9.94 ± 4.35% and that of CD56^+^ cells from 1.19 ± 0.35 to 6.13 ± 3.9% (*p* < 0.05) (Figure 1B). A similar upregulation of the receptors was observed after an incubation of PBL with recombinant Hsp70 protein instead of TKD peptide: (CD94^+^ cells: 5.55 ± 1.65; CD69^+^ cells: 11.58 ± 4.38; CD56^+^ cells: 6.72 ± 4.75) (Figure 1B). Concomitantly, the mean fluorescence intensities of CD94, CD56 which serve as surrogate markers for the Hsp70-specificity increased significantly on CD3-negative NK cells compared to unstimulated control cells (Figure 1C). No significant changes in activation markers were observed on CD3^+^ T cell population upon stimulation with TKD/IL-2 or Hsp70/IL-2 (*data not shown*).

### *Ex vivo* TKD/IL-2-Stimulated NK Cells Combined With Anti-PD-1 Antibody Demonstrate Enhanced Cytotoxic Activity Toward Tumor Cells

**Figure 2:**
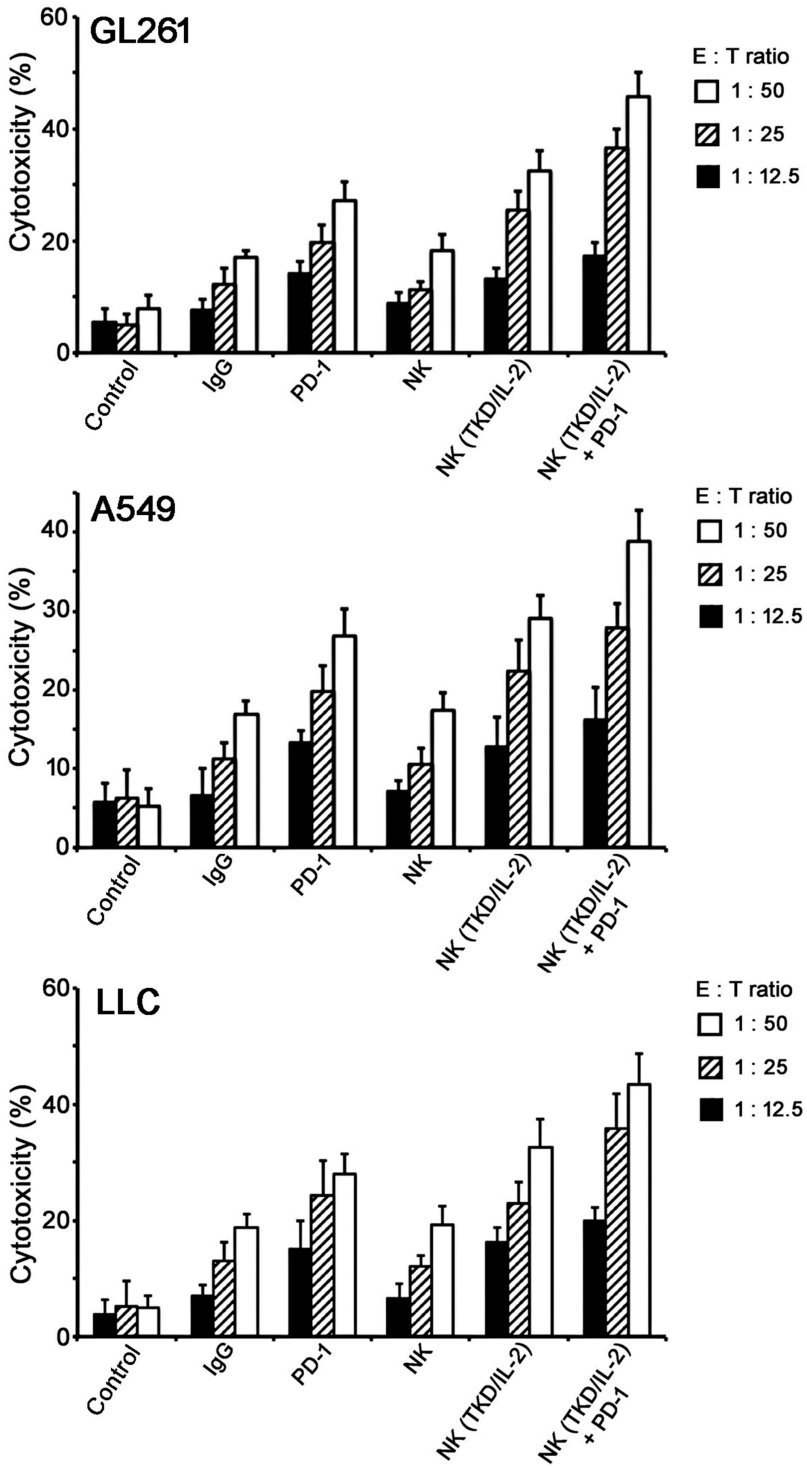
Cytolytic activity of the *ex vivo* stimulated NK cells with anti-PD-1 antibody toward GL261, A549, and LLC tumor cells. Data presented as means ± standard error (M ± SE) for three independent experiments.

To assess the effect of a combined application of TKD/IL-2-stimulated NK cells with anti-PD-1 antibody *in vitro*, tumor cells (GL261, A549, and LLC) were co-incubated with activated lymphocytes at various effector:target (E:T) cells ratios ranging from 1:50 to 1:12.5. To prove that NK cell activity is measured in the assay the lysis of the NK target cell line K562 was assessed. The lysis of K562 cells at an E:T ratio of 1:50 was 20, 34, and 55% by unstimulated NK cells, NK cells stimulated with TKD/IL-2, and NK cells stimulated with TKD/IL-2 plus PD-1 antibody, respectively. With respect to the tumor cell lines GL261, A549, and LLC a co-incubation of unstimulated PBL with species-specific PD-1 antibody resulted in a more than two-fold increase in the lysis of all tumor cells (Figure 2). This effect was comparable to that of a stimulation of mouse and human PBL with TKD/IL-2. The most prominent anti-tumor cytolytic activity was achieved when PBL were stimulated with TKD/IL-2 concomitant with anti-PD-1 antibody (*p* < 0.001).

### Treatment With *ex vivo* TKD/IL-2-Activated, Mouse NK Cells, and Anti-PD-1 Antibody Significantly Enhances OS and Induces Immune Cell Infiltration in a Syngeneic Glioblastoma Mouse Model

**Figure 3:**
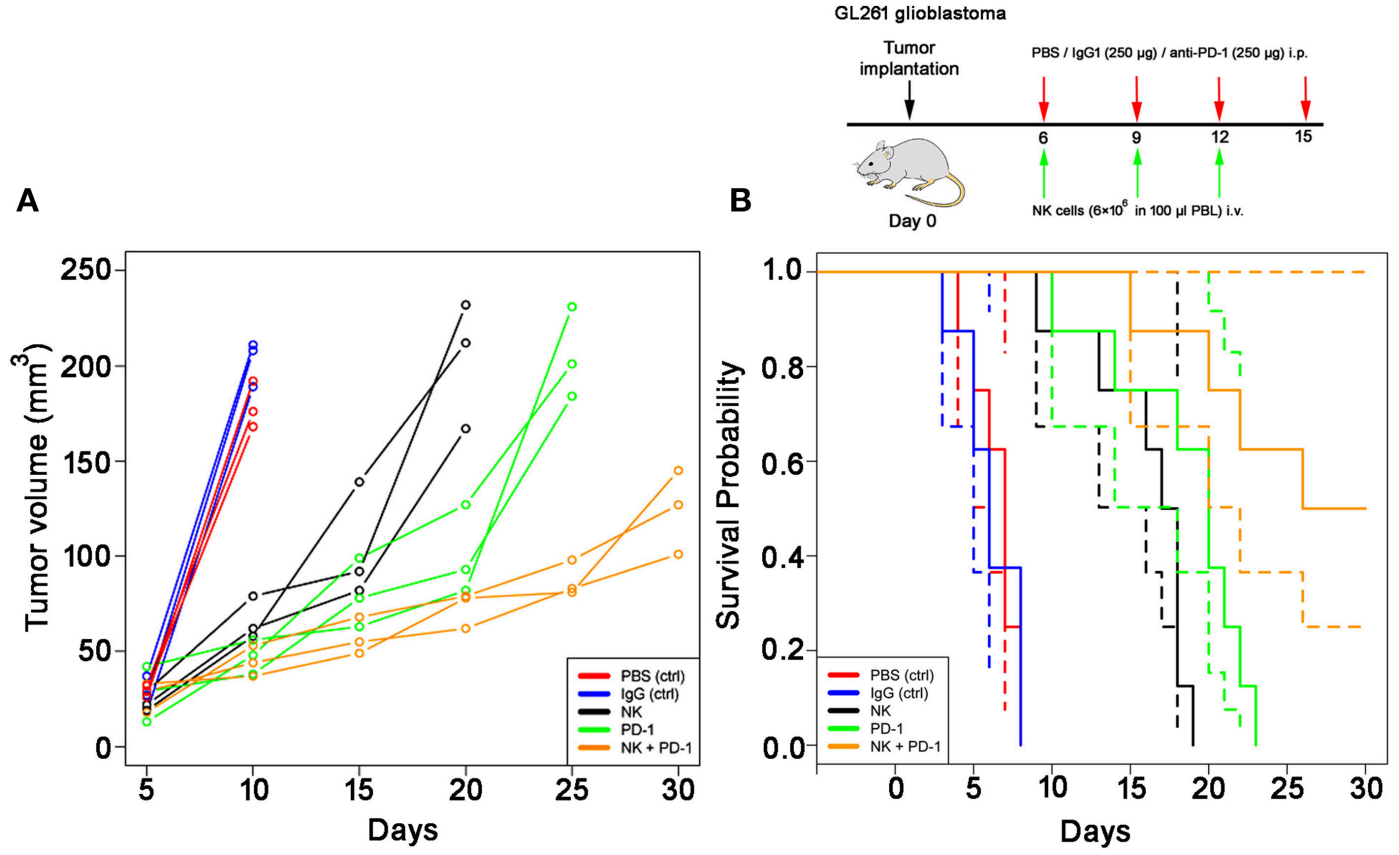
Therapeutic potency of a combined treatment with *ex vivo* TKD/IL-2- stimulated, mouse NK cells with anti-PD-1 antibody in the model of intracranial GL261 glioma. **(A)** Volumetric studies of the GL261 glioma. Tumor volume (mm^3^) as determined over time by T_1_-weighted and T_2_-weighted MRI scans in control (PBS, red lines; IgG control antibody, black lines) and treated (NK cells, green lines; PD-1 antibody, orange lines; NK cells + PD-1 antibody, blue lines) glioblastoma (GL261)-bearing C57/Bl6 mice (*n* = 3 per group). Tumor progression was calculated by measuring the cross-sectional areas on each slice and multiplying their sum as related to the thickness of the sections. **(B)** Kaplan-Meier analysis of the cumulative survival (days after treatment) in control (PBS, red lines; IgG control antibody, blue lines) and treated (NK cells, black lines; PD-1 antibody, green lines; NK cells + PD-1 antibody, orange lines) glioblastoma (GL261)-bearing C57/Bl6 mice (*n* = 8 per group). Solid lines: mean values; dotted lines: SD within 95% confidence interval.

The effects of a singular or combined treatment consisting of *ex vivo* TKD/IL-2-stimulated mouse effector cells (NK) and immune checkpoint inhibitor blockade against mouse PD-1 (PD-1) were determined in mice with membrane Hsp70 positive orthotopic glioblastomas (GL261) (22). The treatment was started when the tumors reached a size of 100 mm^3^ approximately on day 6. The most rapid tumor growth was observed in sham-treated (PBS, IgG isotype-matched antibody) control mice, as determined by MRI scanning (Figure 3A). On day 10, tumors reached a volume of 179 ± 12 mm^3^ (PBS) and 203 ± 12 mm^3^ (IgG, Table 1), and all mice of the control groups died before day 15 (Figure 3B). Three iv injections of *ex vivo* TKD/IL-2-activated NK cells, or 4 ip injections of mouse anti-PD-1 antibody caused a significant tumor growth delay. The maximum tumor volume of 203 ± 33 and 205 ± 24 mm^3^ was reached 10 and 15 days later than in the sham-treated control group (Table 1). The best therapeutic outcome was achieved after a combined treatment with *ex vivo* mouse NK cells and PD-1 antibody. Even on day 30, the size of the tumors of 4 mice was only 124 ± 22 mm^3^, and 4 out of 8 mice treated with the combined therapeutic approach showed complete tumor control (Table 1).

**Table 1:** Tumor volumes (mm^3^) of mice (*n* = 8 per group) of control (ctrl) and treatment groups (NK, PD-1, NK + PD-1).

	**Day 5**	**Day 10**	**Day 15**	**Day 20**	**Day 25**	**Day 30**
PBS (ctrl)	29 (3)	178.67 (12.22)				
IgG (ctrl)	27.33 (9.50)	202.67 (11.93)				
NK	23.33 (5.13)	66.33 (11.15)	104.33 (30.43)	203.67 (33.29)		
PD-1	28 (14.52)	47.33 (9.02)	80 (18.08)	100.67 (23.46)	205.33 (23.80)	
NK + PD-1	26.67 (7.77)	44.66 (8.02)	57.33 (9.71)	73 (9.54)	87.33 (9.29)	124.33 (22.12)

*Sham treatment: PBS (ctrl, 100 μl, iv), IgG (ctrl, 500 μl, ip) isotype-matched control antibody; treatment: NK, ex vivo TKD/IL-2-activated NK cells (6x10^6^ cells in 100 μl PBS, iv); PD-1, PD-1 antibody (500 μl, ip); NK + PD-1, ex vivo activated NK cells (6x10^6^ cells in 100 μl PBS, iv) + PD-1 (500 μl, ip) antibody over 6 time points (days 5, 10, 15, 20, 25, 30). The data represent mean values ± SD*.

**Figure 4:**
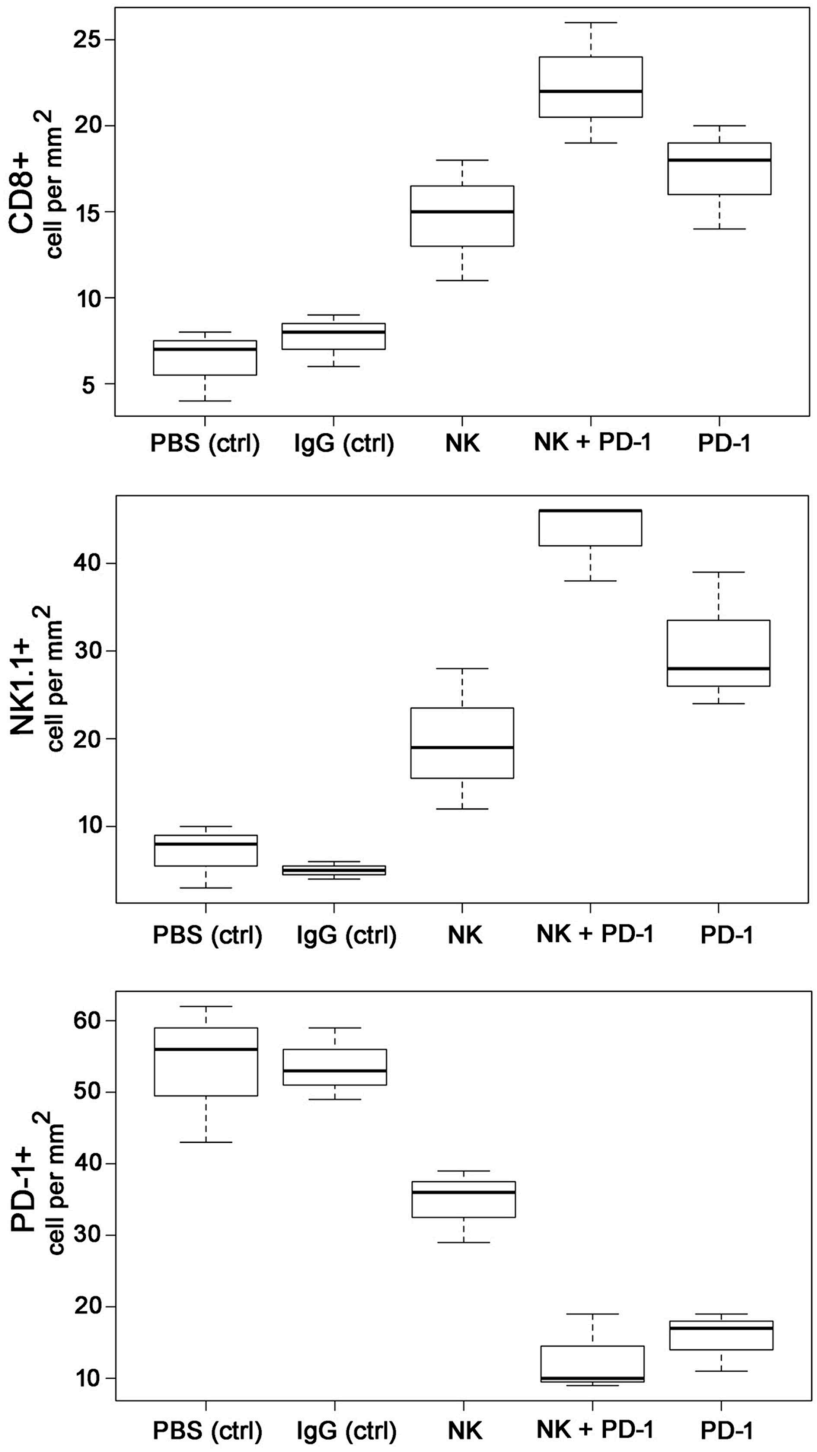
Boxplots of the CD8+, NK1.1+ and PD-1+ cells infiltrating the GL261 tumor in control (PBS and IgG treated animals) and experimental groups. Tumor infiltrating CD8+ cytotoxic T cells, CD3-/CD56+ NK cells and PD1+ effector (T/NK) cells were counted in three representative IHC sections of the tumors of the different treatment groups (PBS ctrl, IgG ctrl, NK, NK + PD-1, PD-1) and infiltrating immune cells per mm^2^ were calculated.

As shown by Kaplan-Meier analysis, OS of mice treated either with NK cells (3 injections, iv) or anti-PD-1 antibody (4 injections, ip) was significantly (*p* < 0.05) higher than that of sham-treated mice (PBS, 3 injections, iv; IgG, 4 injections, ip) (Figure 3B, Table 2). The *p*-values constituted *p* < 0.0001 for both, NK cell and anti-PD-1-treated groups vs. control. It appeared that 4 treatment cycles with mouse anti-PD-1 antibody were not significantly different regarding the OS as compared to the animals treated with three cycles of pre-activated NK cells (*p* = 0.22). Due to the iv route, the number of NK injections was limited to three cycles. The best therapeutic outcome was observed in mice after a combined treatment. OS of these mice was significantly higher than that of the sham-treated control groups (*p* < 0.00001) and that of NK or PD-1 antibody treated mice (*p* < 0.00001). In line with these findings, the number of tumor-infiltrating CD8^+^ T and NK1.1 cells in tumor sections of mice, treated with NK cells and PD-1 antibody was significantly higher than in the control group (*p* < 0.01), and in the group of mice treated either with NK cells or PD-1 antibody (*p* < 0.05; Figure 4, Table 3). Vice versa, the number of tumor-infiltrating effector cells expressing the immune checkpoint inhibitor PD-1 decreased significantly (*p* < 0.001) in the treatment groups (PD-1, NK, NK + PD-1).

**Table 2:** Means and standard deviations (SD) of survival in days for mice with orthotopic GL261 glioblastoma subjected to different treatment and control regimes.

	**PBS (Ctrl)**	**IgG (Ctrl)**	**NK**	**PD-1**	**NK + PD-1**
Mean	11.5	11.1	21	23.5	29.9
SD	1.41	1.8	3.4	4.4	5.3

**Table 3:** Number of tumor-infiltrating CD8^+^ T cells, NK1.1 cells and PD-1^+^ expressing effector cells in tumor sections of mice of the sham-treated control (ctrl) and treatment groups (NK, PD-1, NK+PD-1).

	**NK 1.1 cells**	**CD8 cells**	**PD-1^+^ cells**
PBS (ctrl)	7 (3.61)	6.33 (2.08)	53.67 (9.71)
IgG (ctrl)	5 (1)	7.67 (1.53)	53.67 (5.03)
PD-1	30.33 (7.77)	17.33 (3.06)	16.67 (4.16)
NK	19.67 (8.02)	14.67 (3.51)	34.67 (5.13)
NK + PD-1	40.33 (4.62)	22.22 (3.51)	12.67 (5.51)

*Sham-treated groups: PBS (ctrl), PBS (100 μl, iv), IgG (ctrl), isotype-matched IgG control antibody (500 μl, ip). Treated groups: NK, ex vivo activated NK cells (6x10^6^ cells in 100 μl PBS, iv), PD-1, PD-1 antibody (500 μl, ip), NK+PD-1, ex vivo activated NK cells (6x10^6^ cells in 100 μl PBS, iv) + PD-1 antibody (500 μl, ip). The data represent mean values of three fields of view ± SD*.

### Treatment With *ex vivo* TKD/IL-2-Activated, Human NK Cells, and Anti-PD-1 Antibody Significantly Enhances OS in a Xenograft Lung Carcinoma Mouse Model

**Figure 5:**
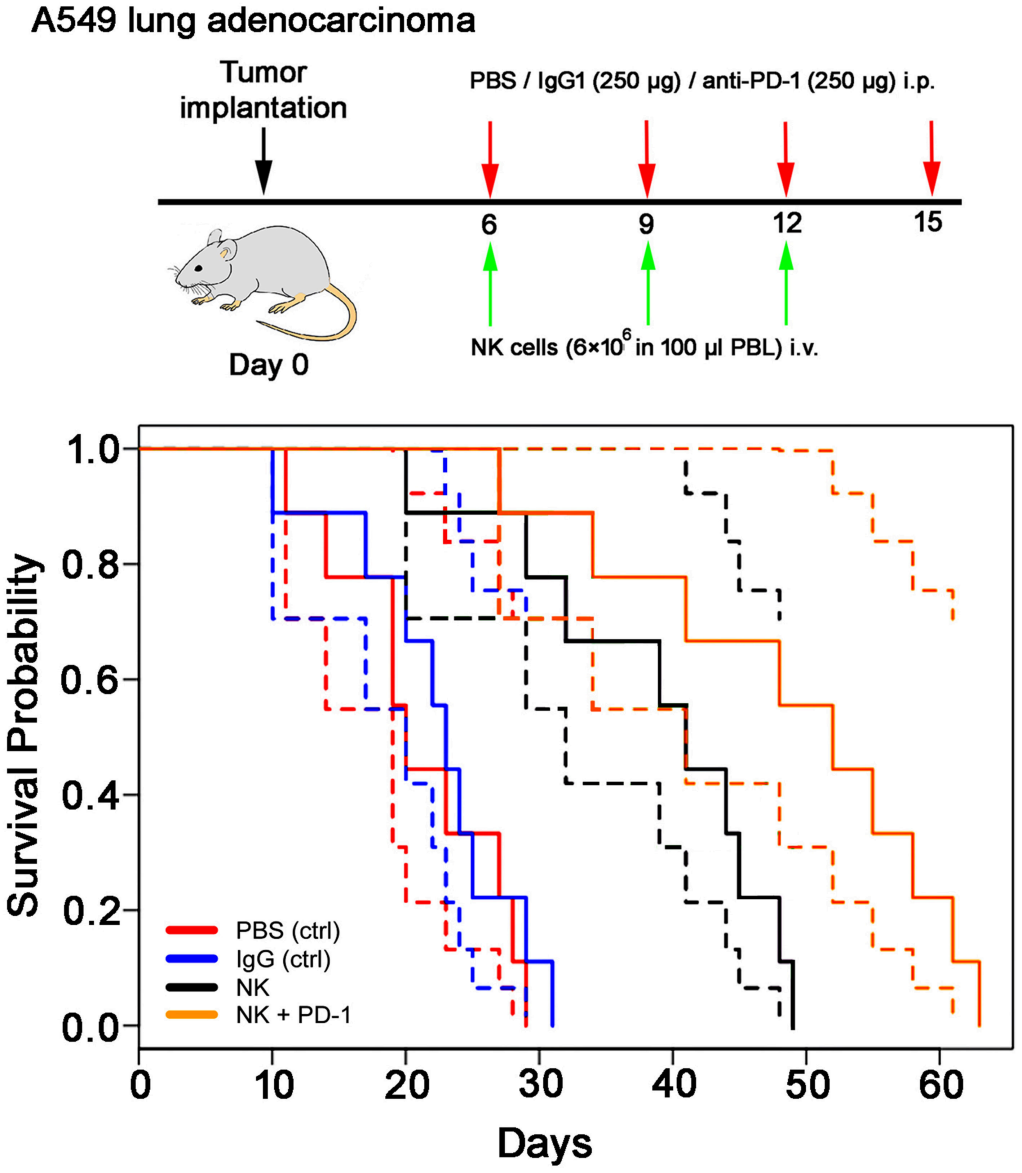
Therapeutic effect of *ex vivo* stimulated, human NK cells with anti-PD-1 antibody in the orthotopic xenograft model of A549 lung carcinoma. Kaplan-Meier analysis of the cumulative survival (days after treatment) in control (PBS, red lines; IgG control antibody, blue lines) and treated (NK cells, black lines; NK cells + PD-1 antibody, orange lines) lung cancer (A549)-bearing mice (*n* = 8 per group). Solid lines: mean values; dotted lines: SD within 95% confidence interval.

**Figure 6:**
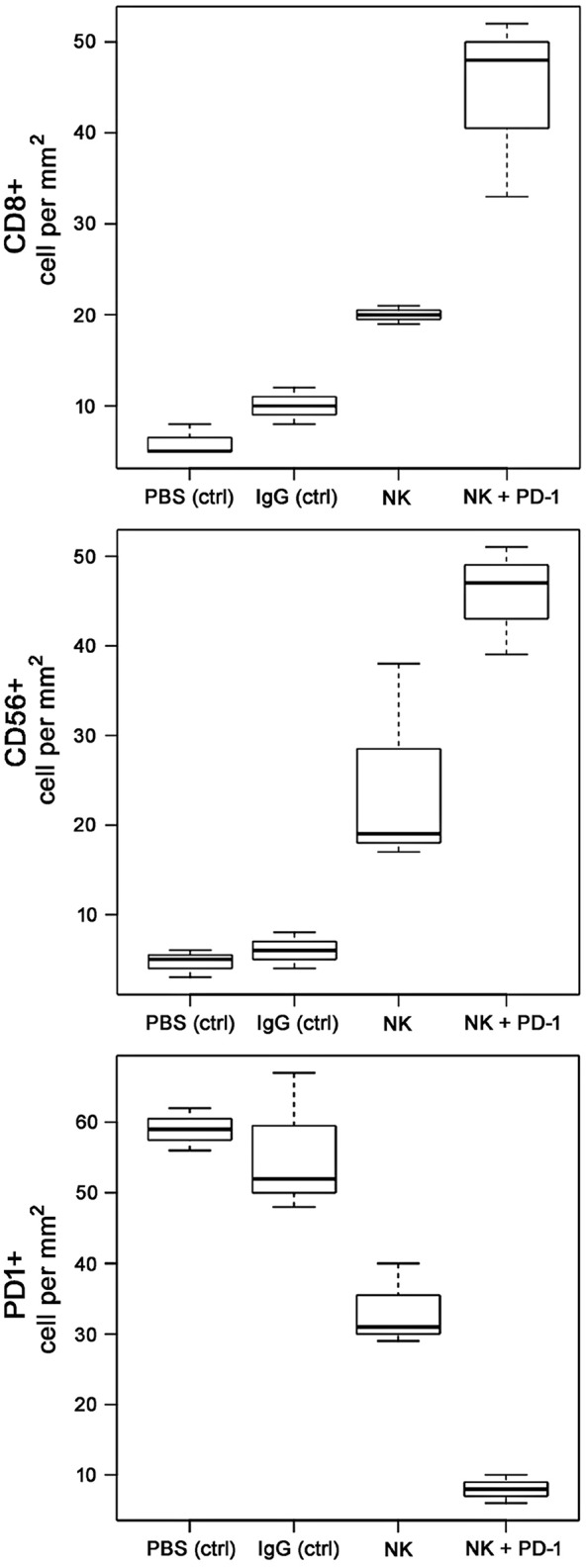
Boxplots of the CD8+, CD56+NK, and PD-1+ cells infiltrating the A549 lung carcinoma in control (PBS and IgG treated animals) and experimental groups. Tumor infiltrating CD8+ cytotoxic T cells, CD3-/CD56+ NK cells, and PD1+ effector (T/NK) cells were counted in three representative IHC sections of the tumors of the different treatment groups (PBS ctrl, IgG ctrl, NK, and NK + PD-1) and infiltrating immune cells per mm^2^ were calculated.

Following iv injection of *ex vivo* TKD/IL-2-stimulated, human effector cells (38.6 ± 9.7 days) a significant increase in the OS of tumor-bearing animals was observed compared to sham (PBS or IgG control antibody) treated control animals (Figure 5, Table 4). A combination of the NK cell therapy and the humanized anti-PD-1 antibody showed a 2.3-fold increase in OS as compared to control animals 48.8 ± 12.4 (NK) and 21.2 ± 6.2 (PBS), 22.3 ± 6.3 (IgG) days, respectively (*p* < 0.001) (Figure 5). Subsequent IHC analysis of the tumor sections showed an increased infiltration by CD56^+^ NK cells and CD8^+^ cells in the treatment groups with a highest infiltration of immune effector cells in the group who received the combined treatment regimen (Figure 6, Table 5). Furthermore, a significant decrease in PD-1^+^ effector cells was observed inside the tumor (*p* < 0.01), as shown by IHC analysis.

**Table 4:** Means and standard deviations (SD) of survival in days for mice with orthotopic lung A549 adenocarcinoma subjected to different treatment and control regimes.

	**PBS (Ctrl)**	**IgG (Ctrl)**	**NK**	**NK + PD-1**
Mean	21.2	22.3	38.6	48.8
SD	6.2	6.3	9.7	12.4

**Table 5:** Number of tumor-infiltrating CD8+ T cells, CD56+ NK cells and PD-1+ expressing effector cells in A549 lung carcinoma sections of mice of the sham-treated control (ctrl) and treatment groups (NK, PD-1, NK+PD-1).

	**Cell type**		
**Treatment**	**CD56+ NK cells**	**CD8+ cells**	**PD-1+ cells**
PBS (ctrl)	4.67 (1.53)	6.00 (1.73)	59.00 (3.00)
IgG (ctrl)	6.00 (2.00)	10.00 (2.00)	55.67 (10.02)
NK	24.67 (11.59)	20.00 (1.00)	33.33 (5.86)
NK + PD-1	45.67 (6.11)	44.33 (10.02)	8.00 (2.00)

*Sham-treated groups: PBS (ctrl), PBS (100 μl, iv), IgG (ctrl), isotype-matched IgG control antibody (500 μl, ip). Treated groups: NK, ex vivo activated NK cells (6 × 10^6^ cells in 100 μl PBS, iv), ex vivo activated NK cells (6 × 10^6^ cells in 100 μl PBS, iv) + PD-1 antibody (500 μl, ip). The data represent mean values of three fields of view ± SD*.

## Discussion

Immune checkpoint inhibitors directed against CTLA-4, PD-1 or PD-L1 have recently demonstrated a therapeutic benefit in various solid tumors (e.g., melanoma, head and neck squamous cell carcinoma, gastric cancer, colorectal cancer, NSCLC, etc.) and lymphoid malignancies (26–31). Recently, evidence has accumulated that combined therapeutic strategies

that consist of several immune checkpoint inhibitors or immune checkpoint inhibitors and other treatment modalities (32, 33) are beneficial. In the presented study anti-PD-1 immune checkpoint antibodies were combined with a NK cell therapy in a syngeneic and xenograft tumor mouse model. As shown previously, a blockade of immune checkpoints could improve NK cell-based therapies (34). Guo et al. demonstrated that anti-PD-1 antibody significantly increased the cytotoxicity of NK cells (i.e., enhanced expression of NKp30, NKp44 and NKG2D) that resulted in therapeutic effect toward multiple myeloma cells (35). Subsequent studies proved combined effect of Pidilizumab (anti-PD-1) either alone or in combination with Rituximab in facilitation of the cytolytic activity of NK cells in patients with follicular lymphoma, multiple myeloma and renal cell carcinoma (36, 37).

In series of *in vitro* experiments for analysis of NK cells cytolytic activity toward tumor cells (GL261, A549, LLC) we demonstrated the therapeutic potential of a monotherapy when *ex vivo* TKD/IL-2-activated NK cells were applied (Figure 2). The effect was significantly higher as compared to non-stimulated lymphocytes. TKD/IL-2 activation of NK cells upregulated expression of CD56, CD69, and CD94 (Figure 1) that subsequently resulted in an enhanced cytotoxicity of lymphocytes (38). Previously, Gross et al. demonstrated that an increased expression density of CD94/NKG2C and CD56 initiates the NK cells capacity to kill membrane Hsp70-positive tumor cells (39, 40) and thereby acts as a surrogate marker for Hsp70-reactivity. The observed cytolytic effect of TKD/IL-2-stimulated NK cells was comparable to that of lymphocytes which have been pre-incubated with anti-PD-1 monoclonal antibodies (Figure 2). Previously, it was shown that blockade of PD-1 on NK cells could improve the cytotoxicity of the lymphocytes (even of exhausted NK cells in advanced tumor stages) (36, 41). A combination of TKD/IL-2-stimulated NK cells with anti-PD-1 antibodies resulted in 1.5-fold increase of anti-tumor cytotoxicity of lymphocytes (Figure 2) that indicates the synergistic effect of both therapeutic concepts.

In our experiments a preclinical proof-of-principle study has shown promising results of a combined therapy consisting of *ex vivo* TKD/IL-2-stimulated NK cells and anti-PD-1 antibody with respect to local tumor control, OS and immune stimulation in immunocompetent and immunodeficient mice with membrane Hsp70-positive tumors (GL261 glioblastoma, A549 lung cancer). The observed therapeutic efficacy was comparable to the effects reported earlier (42–44). Intriguingly, a significantly improved OS was observed when NK cell therapy was combined with anti-PD-1 antibody in a syngeneic GL261 glioblastoma and a xenograft A549 lung cancer model. Previously it was reported that cancer types (including NSCLC and melanoma), which are most responsive to checkpoint inhibitors, have a high mutational load (45, 46). Anti-tumor responses in mice were accompanied by a massive infiltration of the tumors with CD8^+^ cytotoxic lymphocytes and NK1.1 cells, and a reduction in the amount of PD-1^+^ immune cells in the tumor. Although NK cells or anti-PD-1 antibody, as a single treatment modality, have been shown to trigger anti-tumor immune responses that increase OS, a combined therapy has been found to be significantly more efficient. Presumably this could be explained by the effect of the anti-PD-1 antibody on NK cells. Programmed death 1 (PD-1) receptor was originally determined as an exhaustion marker on T cells, however, this receptor is also expressed on NK cells. In the recent study by Concha-Benavente et al. it was shown that PD-1 blockade increased Cetuximab-mediated NK cell activation and cytotoxicity in the head and neck patients (47). The anti-tumor effect achieved by monotherapies (i.e., TKD/IL-2-stimulated NK cells or anti-PD-1 antibodies) that resulted in the delayed tumor progression (Figure 3A) was shortly abrogated after the discontinuation of the therapies. However, combined treatment approaches demonstrated the sustainability of the therapeutic effect after the discontinuation. Presumably, to further potentiate the therapeutic benefit a long-term combinatorial immunotherapy should be considered.

In our study we employed the inhibitor of PD-L1/PD-1 axis for the enhancement of NK cell adoptive therapy. Recently other immune checkpoint inhibitors (e.g., anti-CTLA-4 antibodies, anti-NKG2A antibodies) have been reported to restore cytolytic functions of NK cells and thereby enhance their anti-tumor activity (48, 49). Thus, André et al. showed that humanized anti-NKG2A antibodies enhanced NK cell activity against various tumor cells and rescued CD8^+^ T cell function (49). Presumably, combination of TKD/IL-2-stimulated NK cells with several therapeutic antibodies could improve the anti-tumor activity of the adoptive cell immunotherapies.

Depending on its subcellular or extracellular localization, Hsp70 fulfills different functions (50). On the one hand membrane Hsp70 serves as a tumor-specific target for TKD/IL-2-activated NK cells (4, 51), on the other hand, high cytosolic Hsp70 levels can interfere with apoptotic pathways that mediate radio-chemotherapy resistance. However, as was shown previously the upregulation of the membrane-bound Hsp70 following anti-tumor therapies (e.g., ionizing radiation, chemotherapy, etc.) also increases the efficacy of the Hsp70-targeted therapies (52, 53).

In line with the data shown in two preclinical models most recently, we could demonstrate the efficacy of the combined therapeutic concept consisting of radiochemotherapy, TKD/IL-2-activated NK cells and PD-1 inhibition in a patient with membrane Hsp70 positive stage IIIb NSCLC. Identical to the mouse models, the therapy was well tolerated, induced anti-tumor immune responses mediated by T and NK cells and resulted in a long-term OS of more than 35 months (54).

In summary our data indicate that immunotherapeutic approaches with minor monoactivity could be enhanced by the addition of immune checkpoint inhibitors. The efficacy of a combined therapy consisting of *ex vivo* stimulated NK cells and anti-PD-1 blockade which has been shown to be feasible, safe, and effective needs to be validated in randomized clinical trials.

## Author Contributions

The study was conceived and designed by MS and GM. MS, EP, AI, SS, WK, OG, SE, DL conducted and analyzed the experiments. EP supported statistical analysis. MS and GM wrote the manuscript with valuable comments from DL, SS, WK, OG, and AI.

### Conflict of Interest Statement

The authors declare that the research was conducted in the absence of any commercial or financial relationships that could be construed as a potential conflict of interest.
